# Classification of stomach adenocarcinoma based on fatty acid metabolism-related genes frofiling

**DOI:** 10.3389/fmolb.2022.962435

**Published:** 2022-08-26

**Authors:** Chunhua Liu, Yongjun Tao, Huajian Lin, Xiqiang Lou, Simin Wu, Liping Chen

**Affiliations:** ^1^ Department of Tumor Rehabilitation Center, Lishui Hospital of Traditional Chinese Medicine, Affiliated to Zhejiang University of Traditional Chinese Medicine, Lishui, China; ^2^ Research Center of Lishui Hospital of Traditional Chinese Medicine, Affiliated to Zhejiang University of Traditional Chinese Medicine, Lishui, China

**Keywords:** fatty acid metabolism, stomach adenocarcinoma, tumor immune microenvironment, immune checkpoint inhibitors therapy, prognosis

## Abstract

**Background:** Fatty acid metabolism (FAM)-related genes play a key role in the development of stomach adenocarcinoma (STAD). Although immunotherapy has led to a paradigm shift in STAD treatment, the overall response rate of immunotherapy for STAD is low due to heterogeneity of the tumor immune microenvironment (TIME). How FAM-related genes affect TIME in STAD remains unclear.

**Methods:** The univariate Cox regression analysis was performed to screen prognostic FAM-related genes using transcriptomic profiles of the Cancer Genome Atlas (TCGA)-STAD cohort. Next, the consensus clustering analysis was performed to divide the STAD cohort into two groups based on the 13 identified prognostic genes. Then, gene set enrichment analysis (GSEA) was carried out to identify enriched pathways in the two groups. Furthermore, we developed a prognostic signature model based on 7 selected prognostic genes, which was validated to be capable in predicting the overall survival (OS) of STAD patients using the univariate Cox regression, least absolute shrinkage and selection operator (LASSO) regression, and multivariate Cox regression analyses. Finally, the “Estimation of STromal and Immune cells in MAlignant Tumours using Expression data” (ESTIMATE) algorithm was used to evaluate the stromal, immune, and ESTIMATE scores, and tumor purity of each STAD sample.

**Results:** A total of 13 FAM-related genes were identified to be significantly associated with OS in STAD patients. Two molecular subtypes, which we named Group 1 and Group 2, were identified based on these FAM-related prognostic genes using the consensus clustering analysis. We showed that Group 2 was significantly correlated with poor prognosis and displayed higher programmed cell death ligand 1 (PD-L1) expressions and distinct immune cell infiltration patterns. Furthermore, using GSEA, we showed that apoptosis and HCM signaling pathways were significantly enriched in Group 2. We constructed a prognostic signature model using 7 selected FAM-related prognostic genes, which was proven to be effective for prediction of STAD (HR = 1.717, 95% CI = 1.105–1.240, *p* < 0.001). After classifying the patients into the high- and low-risk groups based on our model, we found that patients in the high-risk group tend to have more advanced T stages and higher tumor grades, as well as higher immune scores. We also found that the risk scores were positively correlated with the infiltration of certain immune cells, including resting dendritic cells (DCs), and M2 macrophages. We also demonstrated that elevated expression of gamma-glutamyltransferase 5 (*GGT5*) is significantly associated with worse OS and disease-free survival (DFS), more advanced T stage and higher tumor grade, and increased immune cell infiltration, suggesting that STAD patients with high *GGT5* expression in the tumor tissues might have a better response to immunotherapy.

**Conclusion:** FAM-related genes play critical roles in STAD prognosis by shaping the TIME. These genes can regulate the infiltration of various immune cells and thus are potential therapeutic targets worthy of further investigation. Furthermore, GGT5 was a promising marker for predicting immunotherapeutic response in STAD patients.

## Introduction

Stomach adenocarcinoma (STAD) is the fifth most common and third most deadly cancer worldwide (Siegel et al., 2014; Siegel et al., 2019; Smyth et al., 2020). Given the complex pathogenesis and heterogeneity of STAD, early diagnosis and prognostic assessment are extremely difficult, leading to significantly reduced survival in STAD patients (Van Cutsem et al., 2016; Zong et al., 2016; Li et al., 2017). Immunotherapy and targeted therapies, such as anti-programmed cell death ligand 1 (PD-L1) therapy, have shown modest success in improving the outcomes in patients with microsatellite unstable carcinomas (Fuchs et al., 2018). However, the overall therapeutic effect for STAD patients is still unsatisfactory (Shitara et al., 2018). Recent studies have partially elucidated the molecular mechanisms underlying STAD occurrence and development, and identified potential biomarkers, such as circulating CA199, CA724, CEA and CA125, for early screening and prognostic evaluation of STAD. However, these biomarkers have poor sensitivity and specificity and thus are not suitable for individualized treatment and prognosis assessment in clinical practice (Marrelli et al., 1999; Ciliberto et al., 2015; Chau, 2017). Therefore, there is an urgent need to identify effective diagnostic and prognostic biomarkers as well as novel therapeutic targets for STAD diagnosis and treatment.

Metabolic reprogramming is a major feature of malignant transformation (DeBerardinis et al., 2008; Kroemer and Pouyssegur, 2008), enabling the survival, proliferation, and metastasis of cancer cells, even under stressful conditions such as nutrient restriction (Hanahan and Weinberg, 2011; Lunt and Vander Heiden, 2011). The fatty acid metabolism (FAM) pathway is crucial for cancer development and is frequently dysregulated in many types of cancer (Nomura et al., 2010). For instance, the rapidly proliferating tumor cells can utilize these metabolic lipids as an energy source for invasion, metastasis, and neovascularization (Amiri et al., 2018). Menendez et al. showed that lipid synthesis was more active in tumor cells compared to healthy cells, promoting tumor growth (Menendez and Lupu, 2007; Guo et al., 2013). Consistent with this, fatty acid-binding proteins were highly expressed in many types of cancer, such as prostate cancer, breast cancer, and liver cancer, and are associated with cancer metastasis and invasion (Dong et al., 2007). Furthermore, previous studies demonstrated that there is extensive crosstalk between dysregulated metabolic networks and cancer cell signaling, posing a potential new avenue for developing targets and drugs related to cancer metabolism (Vander Heiden et al., 2009; Lunt and Vander Heiden, 2011). Actually, recent studies have shown that FAM-related genes can be used as prognostic markers for some malignant tumors such as breast cancer and lung adenocarcinoma (Balakrishnan et al., 2020; Bogie et al., 2020; Chang and Xing, 2022; Garcia et al., 2022). However, the role of FAM-related genes in STAD progression and prognosis remains unclear.

Changes in specific metabolic pathways also affect immune cell function. For example, the metabolic switching to glycolysis and fatty acid synthesis in macrophages in the tumor microenvironment (TME) can polarize macrophages to the pro-inflammatory phenotype. In addition, glycolysis can activate the interleukin 17 (IL-17)-producing inflammatory T helper cells (Th17) while suppressing the anti-inflammatory regulatory T cells (Tregs) (Buck et al., 2015; Jha et al., 2015). In turn, the accumulation of fatty acids and lipids in the TME can trigger metabolic changes in the tumor-infiltrating immune cells (Jiang et al., 2018). Immune risk score models are useful prognostic tools that can be used to quantify immune cell infiltration in the TME (Duan et al., 2019). These immune scoring systems can improve the predictive accuracy of TNM staging in STAD patients, and thus identify patients with potential better prognoses, which be beneficial in increasing the immunotherapeutic efficacy in these patients (Wang et al., 2020a). It would be promising to explore how FAM-related genes might affect the tumor immune microenvironment (TIME), which might provide novel prognostic biomarkers and therapeutic targets for STAD treatment.

The aim of this study was to assess the prognostic value of FAM-related genes in STAD. Based on the expression levels of FAM-related genes, we identified a total of 13 genes that were significantly associated with overall survival (OS), immunotherapy response and prognosis. We further selected 7 genes to construct a prognostic signature model and a predictive nomogram using the gene risk scores and ages of the patients, which could predict the OS and disease-free survival (DFS) of STAD patients with reasonable accuracy. Furthermore, we showed that *GGT5* is overexpressed in STAD tissues, which is associated with worse prognosis, higher levels of PD-L1 and higher immune cell infiltration. Our findings suggest that expressions of FAM-related genes were prognostically relevant to STAD and can be used to predict patient prognosis and immunotherapeutic response, and guide individual treatment strategies in STAD patients.

## Materials and methods

### Datasets

Based on a previous study (Lee et al., 2020), a list of 309 human FAM-related genes was curated. The most updated (March 2022) transcriptomic profiles and clinical data of the Cancer Genome Atlas (TCGA)-stomach adenocarcinoma (STAD) were downloaded from the Genomic Data Commons Data Portal (https://portal.gdc.cancer.gov/). The expression profiles of 375 STAD tissues and 32 adjacent normal tissues were processed using the PERL software (https://www.perl.org/) and an mRNA expression matrix was generated. The clinical data were similarly processed using the same software and a matrix containing clinical information was generated. Finally, the expression profiles of the 309 FAM-related genes were extracted. The differentially expressed FAM-related genes between the tumor and normal tissues were identified using the “limma” R package, with |log_2_Fold Change| > 1 and *p* < 0.05 set as the cutoffs. According to the guidelines released by the National Cancer Institute in December 2015 (https://cancergenome.nih.gov/publications/publication guidelines), this study does not require ethical approval from the ethics committee.

### Identification of prognostic fatty acid metabolism-related genes

We performed the univariate Cox regression analysis and identified 108 FAM-related genes that were significantly correlated to prognosis in STAD patients. Then, these prognostic FAM-related genes were hierarchically clustered using the “ConsensusClusterPlus” package with K = 2. The “limma” package was used to identify the differentially expressed genes (DEGs) between two groups, and PD-L1 was one of these DEGs. The relationship between the expressions of these DEGs and the clinicopathological parameters was assessed and visualized using the “pheatmap” package. Finally, gene correlation analysis was conducted using the “corrplot” package to clarify the correlation between expressions of PD-L1 and prognostic FAM-related genes in STAD. *p* < 0.05 is regarded as statistically significant.

### Tumor immune microenvironment and gene set enrichment analysis

Tumor purity is inversely proportional to the ratio of infiltrating immune cells (Yin et al., 2022). The relative ratios of infiltrating immune cells in each tumor sample were analyzed using the “preprocessCore”, “limma”, and “e1071” packages. Then we compared the immune cell infiltration between the two groups, and the results were visualized using the violin plots. Gene set enrichment analysis (GSEA) was performed to identify enriched pathways using relative gene sets (c2. Cp.kegg.v.7.2.symbols.gmt, group.cls#G2 versus G1). For GSEA, the number and type of permutations were set at “1,000” and “no Collapse”/“phenotype”, respectively; the gene list ordering mode was set as “descending”; the gene list sorting mode was set as “real”; the metric for ranking genes was set as “Signal2Noise”. *p* < 0.05 is regarded as statistical significant.

### Construction of a prognostic model based on fatty acid metabolism-related genes

The STAD cohort was randomly divided into the training and testing datasets at a 1:1 ratio using the “caret” package. Using the univariate Cox regression, LASSO regression, and multivariate Cox regression analyses, a prognostic risk score model was constructed based on the 7 selected FAM-related genes (Table 1) using the training dataset. The risk score was calculated as following: $\mathrm{risk}\;\mathrm{score}=\mathrm{Coe}f_{\mathrm{MAOA}}\text{×}\mathrm{Expressio}n_{\mathrm{MAOA}}+$ $\mathrm{Coe}f_{\mathrm{PON}1}\text{×}\mathrm{Expressio}n_{\mathrm{PON}1}+\mathrm{Coe}f_{\mathrm{OLAH}}\text{×}\mathrm{Expressio}n_{\mathrm{OLAH}}$ $+\mathrm{Coe}f_{\mathrm{ABCD}1}\text{×}\mathrm{Expressio}n_{\mathrm{ABCD}1}+\mathrm{Coe}f_{\mathrm{THEM}5}\text{×}\mathrm{Expressio}n_{\mathrm{THEM}5}+\mathrm{Coe}f_{\mathrm{GGT}5}\text{×}\mathrm{Expressio}n_{\mathrm{GGT}5}+\mathrm{Coe}f_{\mathrm{ACLY}}\text{×}\mathrm{Expressio}n_{\mathrm{ACLY}}$ , where Coef represents the coefficient with the lowest Akaike’s information criterion (AIC) values. Coef >0 and Coef <0 indicate risk and protective factors, respectively. Based on the median risk score, all the STAD samples were assigned to the high- and low-risk groups. The predictive accuracy of the prognostic model for OS was calculated using the “time-ROC” package. The ROC curves are shown in Figure 4F,I. The risk curves were plotted based on the risk scores and survival status. The independent prognostic factors were further identified by the univariate and multivariate Cox regression analyses. The hazard ratios for these factors were calculated as well. The correlation between the clinical features and risk scores was evaluated. Heatmaps were generated using the “pheatmap” package. Boxplots were used to show the relationship between risk scores and clinical data. The expression levels of PD-L1 in the two groups were also compared. The correlation relationship between expression levels of the 7 FAM-related genes and OS or DFS in STAD patients was analyzed using the Gene Expression Profiling Interactive Analysis (GEPIA) database. The ratios of 22 immune cell types in the high-risk and low-risk groups were determined using the Cell-type Identification by Estimating Relative Subsets of RNA Transcripts (CIBERSORT) algorithm (Newman et al., 2015). The correlations between the risk scores and immune cell scores were analyzed, and scatterplots were generated to show the correlations.

**TABLE 1:** Prognostic signature genes identified from the multivariate Cox regression analysis.

FAM-related genes	HR (95% CI)	*p*-value	Coef
MAOA	1.275 (1.020–1.594)	0.033	0.243
PON1	1.903 (1.055–3.431)	0.032	0.643
OLAH	3.355 (0.934–12.054)	0.064	1.210
ABCD1	0.672 (0.487–0.928)	0.016	−0.398
THEM5	0.623 (0.402–0.965)	0.034	−0.473
GGT5	1.481 (1.093–2.006)	0.011	0.393
ACLY	1.480 (1.135–1.930)	0.004	0.392

FAM, fatty acid metabolism; HR, hazard ratio; CI, confidence interval; Coef, regression coefficient.

### Construction and validation of a predictive nomogram

A prognostic nomogram consisting of the FAM-related gene risk score and clinical indicators was constructed to predict the OS in STAD patients. The constructed nomogram was used to predict the 1-, 3‐ and 5‐year OS, which was compared with the actual OS. Nomogram‐predicted survival and the observed outcome were plotted using the “survminer” package. The prediction accuracy was calculated using the “time-ROC” packages. The 45° line was representative of the best prediction.

### Gene ontology and Kyoto Encyclopedia of Genes and Genomes analyses of differentially-expressed fatty acid metabolism-related genes

The differentially expressed FAM-related genes between the high-risk and low-risk groups were identified using the “limma” package, with |log_2_FC|>1 and *p* < 0.05 set as the cutoffs. The potential functions of these DEGs were analyzed by GSEA. Gene ontology (GO) and Kyoto Encyclopedia of Genes and Genomes (KEGG) analyses were performed using the DAVID (https://david.ncifcrf.gov/) online tool, with *p* < 0.05 as the cutoff. The “clusterProfiler”, “enrichplot”, “org.Hs.eg.db”, “ggplot2” and “GO plot” packages were used for visualization of the results.

### Statistical analysis

R 4.0.4 and Perl 10.0 were used for all statistical analyses. The OS and DFS curves based on the Kaplan-Meier method were plotted using the ggplot2 package. The difference between the two groups was calculated using the log-rank test. DEGs were identified using the “limma” package, and hierarchical clustering analysis was performed using the “ConsensusClusterPlus” package. Univariate Cox regression, LASSO regression and multivariate Cox regression analyses were performed to identify the independent prognostic risk factors, which were used for the construction of a FAM-related prognostic signature.

## Results

### Identification of fatty acid metabolism-related genes related to stomach adenocarcinoma prognosis

**FIGURE 1:**
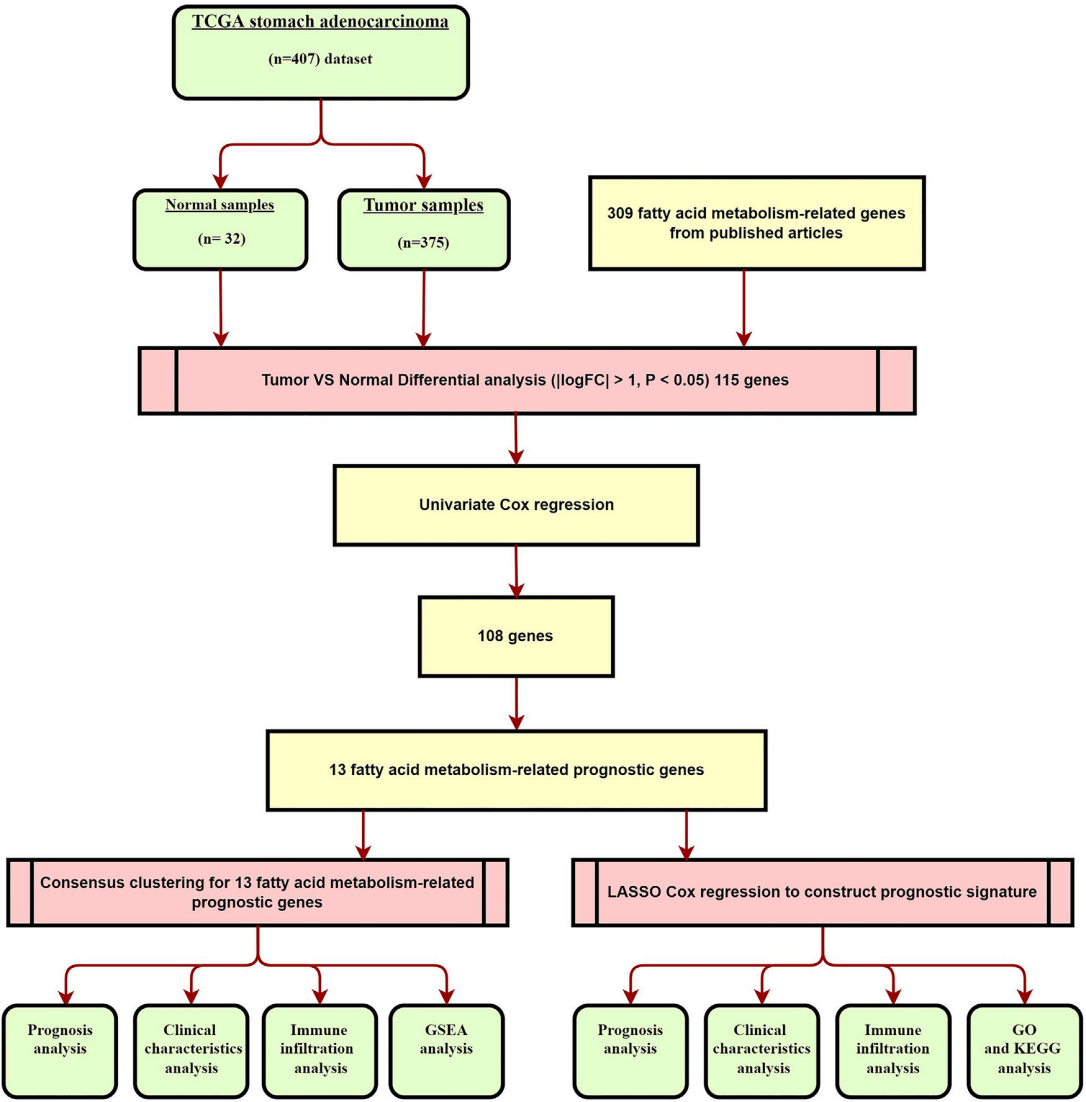
Overview of they study flow chart.

The workflow of the study is shown in Figure 1. In summary, we identified 115 differentially expressed FAM-related genes between the tumor and adjacent normal tissues, with |log_2_FC| > 1 and *p* < 0.05 set as the cutoffs. Among these 115 DEGs, 64 were up-regulated and 51 were down-regulated.

### The biological roles of fatty acid metabolism-related genes in stomach adenocarcinoma

**FIGURE 2:**
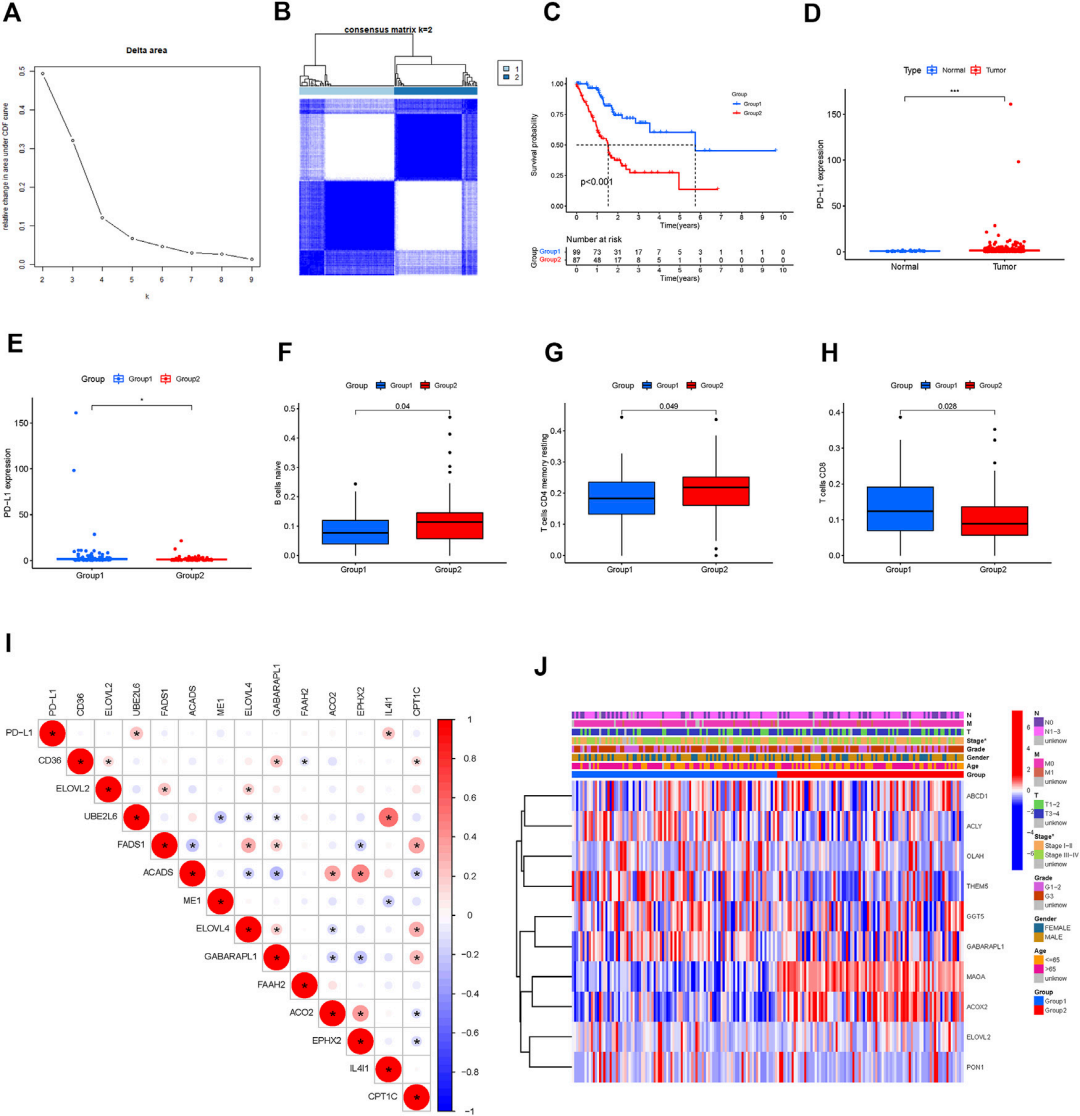
Hierarchical grouping identifies two STAD subtypes based on expression profiling of fatty acid metabolism-related genes. **(A,B)**: grouping analysis. **(C)**: Kaplan-Meier survival analysis illustrated that Group 1 (Immune Response Low) was significantly associated with a better prognosis. **(D**, **E)**: PD-L1 was highly expressed Group 2 compared to Group 1; PD-L1 was highly expressed in STAD tumor tissues compared to adjacent normal tissues **(F,G,H)**: Difference analysis of immune cell infiltration in different groups (Immune cells like B cell naïve, resting memory CD4 T cell were highly clustered in group 2). **(I)**: Correlation analysis of PD-L1 and fatty acid metabolism-genes. **(J)**: Heatmap of differentially expressed prognostic-related genes and relationship with clinicopathological parameters in different clusters. *: *p* < 0.05. Red represents high expression, while blue represents low expression. The abscissa represents the sample, while the ordinate represents prognostic related fatty acid metabolism-related genes.

We identified 108 FAM-related prognostic genes using the univariate Cox regression analysis. The STAD cohort was divided into two distinct groups, which we named as Group 1 and Group2, based on these prognostic genes using the “ConsensusClusterPlus” package with K = 2 (Figures 2A,B). As shown in Figure 2C, patients in Group 2 were significantly associated with a better prognosis (*p* < 0.001). The expressions of the prognostic FAM-related genes were shown using the Heatmap, and the relationship between the expressions of these genes and clinicopathological parameters was plotted as well as shown in Figure 2J. Some of these FAM-related genes were highly expressed in Group 1, whereas others were highly expressed in Group 2. Of note, PD-L1 was highly expressed in Group 2 compared with Group 1, and was highly expressed in tumors compared with adjacent normal tissues, suggesting the two groups might respond differently to immune response (Figures 2D,E). Gene correlation analysis was conducted to ascertain the correlation between PD-L1 and the prognostic fatty acid metabolism-related genes in STAD (Figure 2I). The results showed that PD-L1 is positively correlated to UBE2L6 and IL4I1, which are 2 of these prognostic fatty acid metabolism-related genes (*p* < 0.05). There were 13 differentially expressed genes related to FAM associated with prognosis. We found no significant difference regarding clinical characteristics between the two groups except for tumor stage and N stage.

**FIGURE 3:**
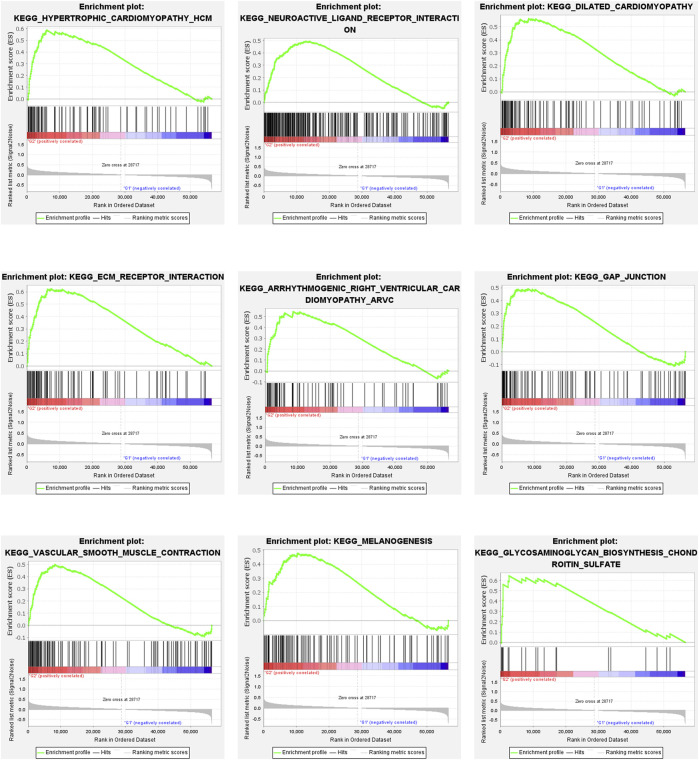
Gene Set Enrichment Analysis. Gene set enrichment analysis (GSEA) showed that tumor hall markers were enriched in the high-risk group. All of them are positively related to G2. Nominal *p*-Value < 0.05.

Immune cell infiltration in the two groups was analyzed. As shown in Figures 2F–H, naïve B cells and resting memory CD4^+^ T cells were predominant in Group 2, while CD8^+^ T cells were predominant in Group 1 (*p* < 0.05). Multiple pathways, including the hypertrophic cardiomyopathy (HCM) pathway, were identified as the most enriched pathway in Group 2 (*p* < 0.05; Figures 3A–I).

### Fatty acid metabolism-related prognostic gene signature

**FIGURE 4:**
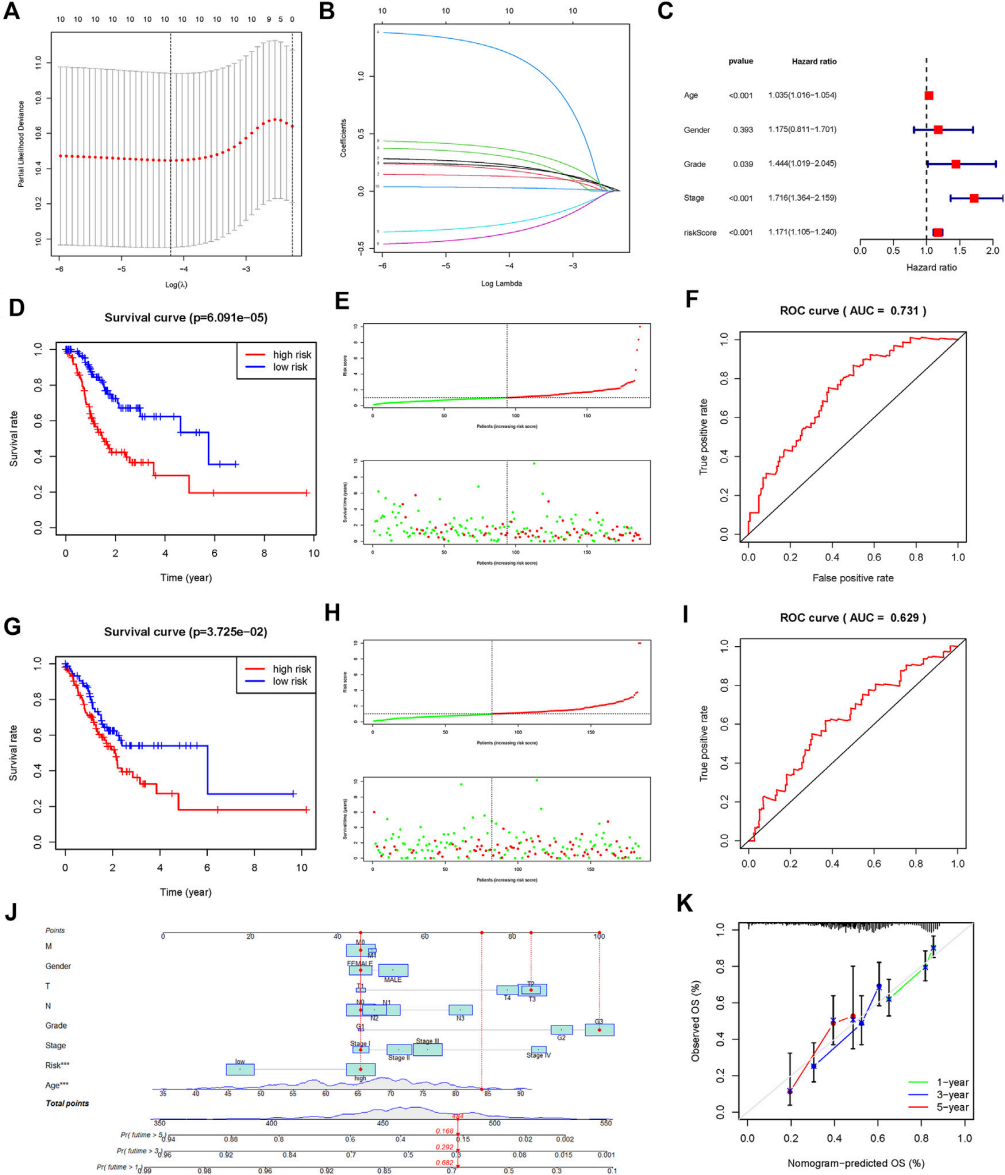
Construction and validation of a prognostic risk signature in TCGA-STAD based on 7 fatty acid metabolism-related genes. **(A,B)**: LASSO regression was performed to identify genes for prognostic model **(C)**: Multivariate analysis of independent prognostic analysis. (Age, Stage and risk score were risk factors for the prognosis of STAD). **(D)**: Kaplan-Meier survival analysis showed that the high-risk group had a poor prognosis and shorter OS in the training dataset. **(E)**: The scatter plot of risk scores in the training dataset. **(F)**: ROC curve to evaluate the accuracy of our model to predict the OS in the training dataset. **(G**–**I)**: The survival plot, scatter plot of risk scores, and roc curve in the testing dataset. **(J)** Nomogram based on the age, clinical feature, and fatty acid metabolism-related signature. **(K)**: Calibration plots of the nomogram for the prediction of overall survival at 1, 3, and 5 years in the TCGA-STAD cohort.

To construct a prognostic FAM-related gene signature for the prediction of OS in STAD patients, we randomly divided the STAD cohort into the training (Supplementary Table S1) and testing (Supplementary Table S2) datasets in a 1:1 ratio. A 7-gene FAM-related signature was identified in the training dataset, and risk scores of all the samples were calculated (Figures 4A,B). Based on the median risk score, all the patients were divided into the high- and low-risk groups. As shown in Figures 4D,G, the OS for patients in the low-risk group was significantly better than that of patients in the high-risk group, in both the training and testing datasets (*p* < 0.05). To evaluate the predictive accuracy of our model, we plotted the ROC curves for both the training and testing datasets (Figures 4F,I). The AUC values for OS in both datasets were >0.5, indicating that the prognostic model could predict the survival of STAD patients with considerable accuracy. A risk curve was generated, and the survival status and risk of FAM-related genes were assessed (Figures 4E–H).

**FIGURE 5:**
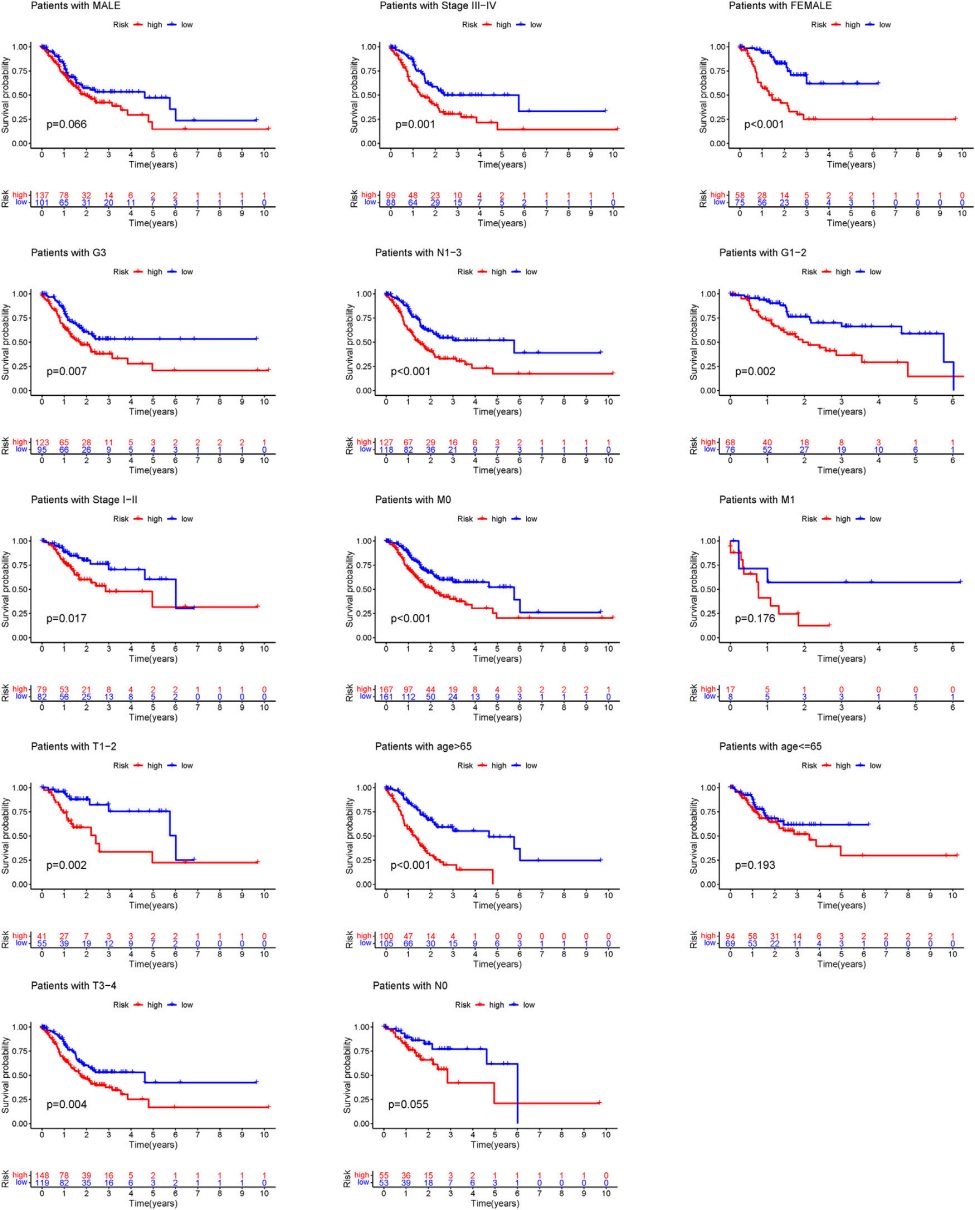
Survival curves for model validation. Our model could be applied to different clinical groups: age, gender, lymph node metastasis, stage and T stage, *p* < 0.05.

Additional prognostic clinical factors for STAD were screened using the univariate Cox regression analysis (Table 2). Furthermore, age, stage and risk score were identified as the independent risk factors using the multivariate Cox regression analysis (Figure 4C, *p* < 0.05). We further constructed a nomogram based on the FAM-related gene signature and age for predicting the prognosis of STAD patients. Predicted OS at 1, 3, and 5 years in STAD patients is shown in Figure 4J. As shown in Figure 4K, the OS predicted from the nomogram is very similar to the actual OS, indicating the nomogram is suitable for clinical application. Furthermore, the signature could be applied to subgroups generated based on age, sex, lymph node metastasis, M stage and T stage (*p* < 0.05; Figure 5).

**TABLE 2:** Univariate and multivariate Cox regression analyses of OS in TCGA-STAD.

Clinicopathologic parameters	Univariate analysis		Multivariate analysis	
	HR (95%CI)	*p*	HR (95%CI)	*p*
Age	1.026 (1.008–1.044)	0.004*	1.035 (1.016–1.054)	<0.001*
Gender	1.249 (0.866–1.802)	0.235	1.175 (0.811–1.701)	0.393
Tumor grade	1.361 (0.969–1.911)	0.075	1.444 (1.019–2.045)	0.039*
Pathologic Stage	1.534 (1.241–1.896)	<0.001*	1.716 (1.364–2.159)	<0.001*
Risk score	1.137 (1.076–1.202)	<0.001*	1.171 (1.105–1.240)	<0.001*

*p < 0.05 was considered statistically significant.

CI, confidence interval; HR, hazard ratio.

**FIGURE 6:**
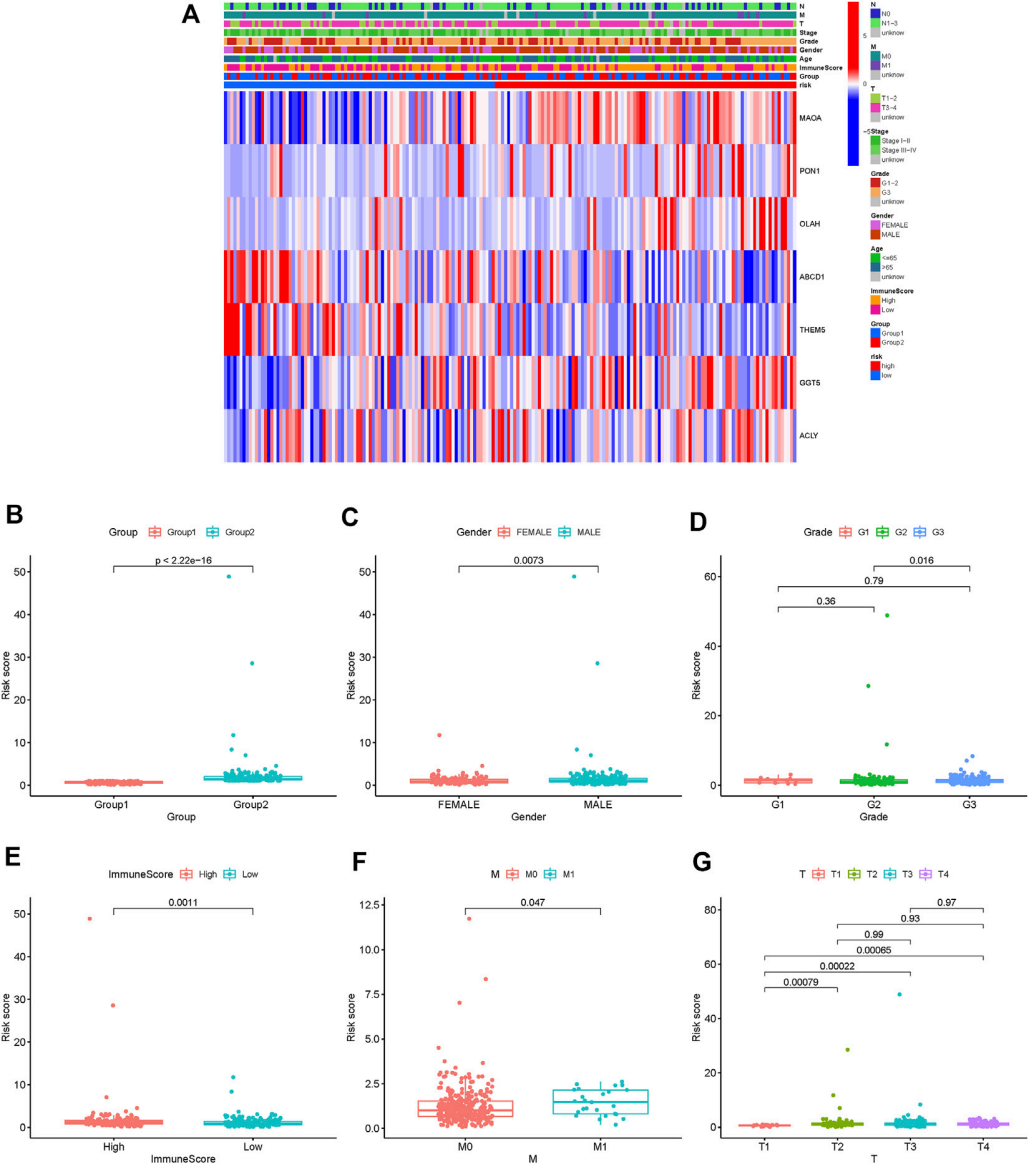
Risk and clinical correlation analysis. **(A)**: Heatmap of risk and clinical correlation analysis; **(B**–**G)**: Boxplot of risk and clinical correlation analysis. (Grade, gender, T stage, M stage, and immune score were closely related to the risk score, *p* < 0.05).

**FIGURE 7:**
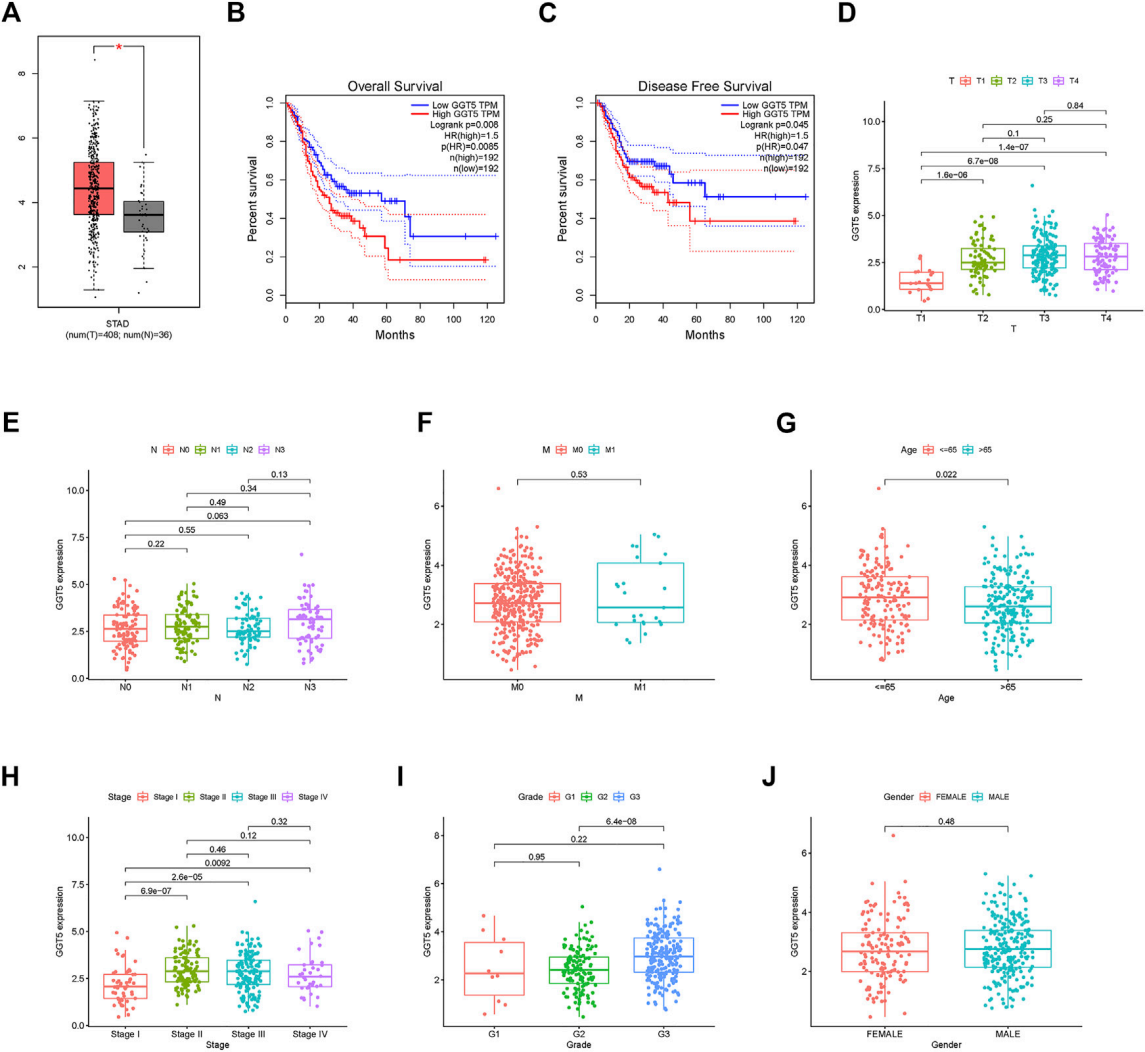
GGT5 expression and clinical correlation analysis. **(A)**: Evaluating GGT5 in the GEPIA database. **(B,C)**: OS plot and DFS plots from the GEPIA database; **(D**–**J)**: Association between GGT5 expression and other clinicopathological features (GGT5 was highly expressed in grade 3, stages II, III, and IV, *p* < 0.05).

To further identify the protective and risk genes in the FAM-related prognostic signature, we analyzed the correlation between the clinical features and expressions of these genes. *MAOA*, *ACLY*, *GGT5*, *OLAH* and *PON1* were highly expressed, while *ABCD1* and *THEM5* were lowly expressed in the high-risk group (Figure 6A, Supplementary Figures S1, S2). Furthermore, tumor grade, N stage, T stage, M stage and gender were significantly correlated to the risk score (Figures 6B–G). We next analyzed the correlations between the expression levels of the FAM-related risk genes OS or DFS. As shown in Figure 7A, compared to adjacent normal tissues, expression of *GGT5* was significantly elevated in STAD tumor tissues. In addition, high expression of *GGT5* was associated with shorter OS and DFS (Figures 7B,C). To assess the correlation between *GGT5* and clinical characteristics, the STAD patients were divided into the *GGT5* ^high^ and *GGT5* ^low^ groups based on the median expression level of *GGT5* (Figures 7D–J). *GGT5* was highly expressed in grade 3 (Figure 7I) and stages II, III and IV tumors (Figure 7H). Furthermore, *GGT5* was highly expressed in patients over 65 years of age with stages T2, T3 and T4 tumors (Figures 7D,G). In contrast, gender, N stage and metastasis were not correlated with the expression of *GGT5*. These findings suggest that *GGT5* is an independent prognostic biomarker for STAD.

### Association between risk score and immune infiltration

**FIGURE 8:**
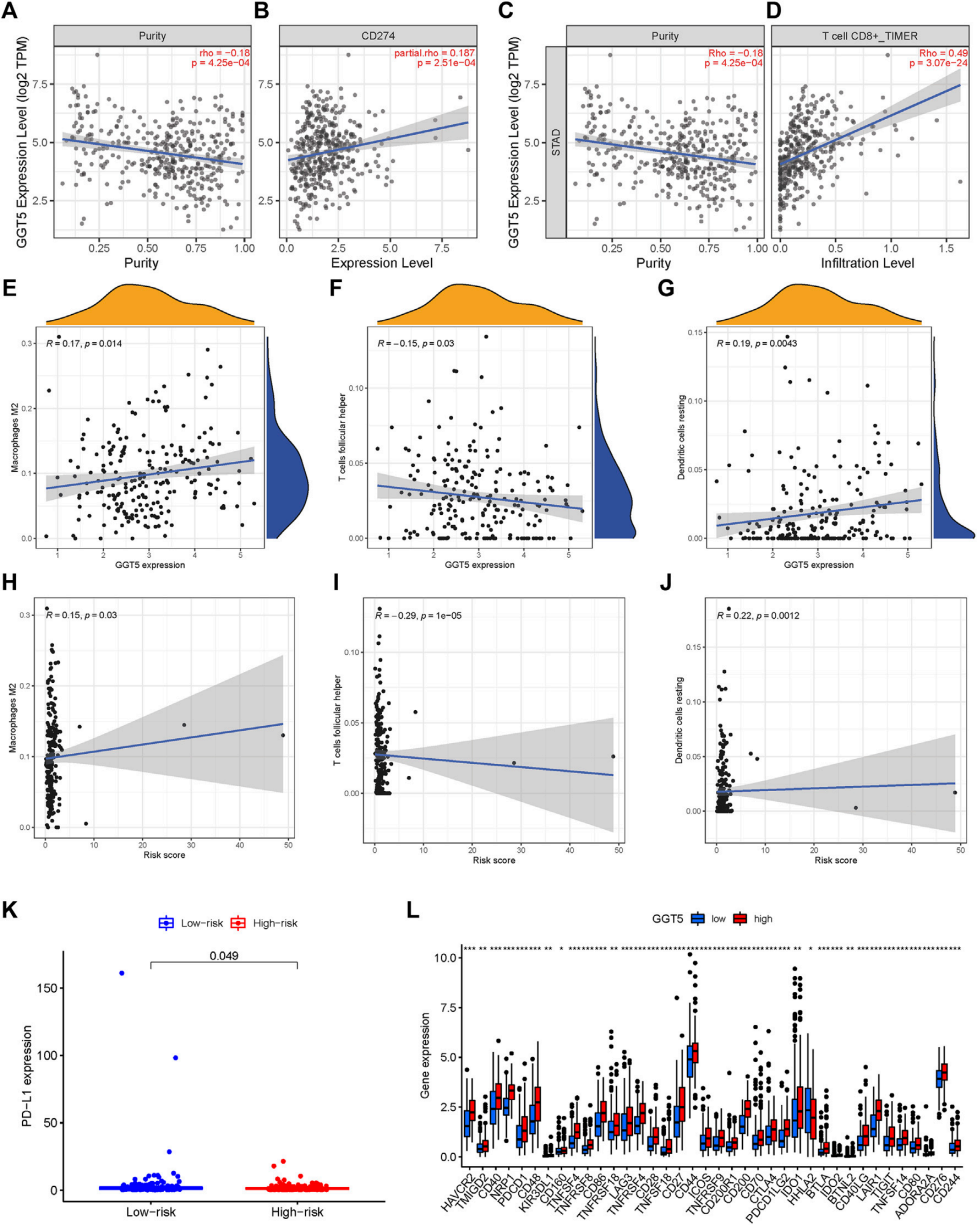
Scatter plots of correlation analysis of risk score and GGT5 expression with immune cells. **(A**–**G)**: scatter plots of correlation analysis of GGT5 expression with PD-L1 and immune infiltration level; **(H**–**J)**: scatter plots of correlation analysis of risk score and immune cells. Both Macrophages M2 and Dendritic cells resting are positively related with the risk score, R > 0 and *p* < 0.05.T cells follicular helper is negatively related with a risk score, R < 0 and *p* < 0.05. **(K–L)**: expression of immune checkpoint genes in high-risk and low-risk groups and their correlation with GGT5 expression levels.

**FIGURE 9:**
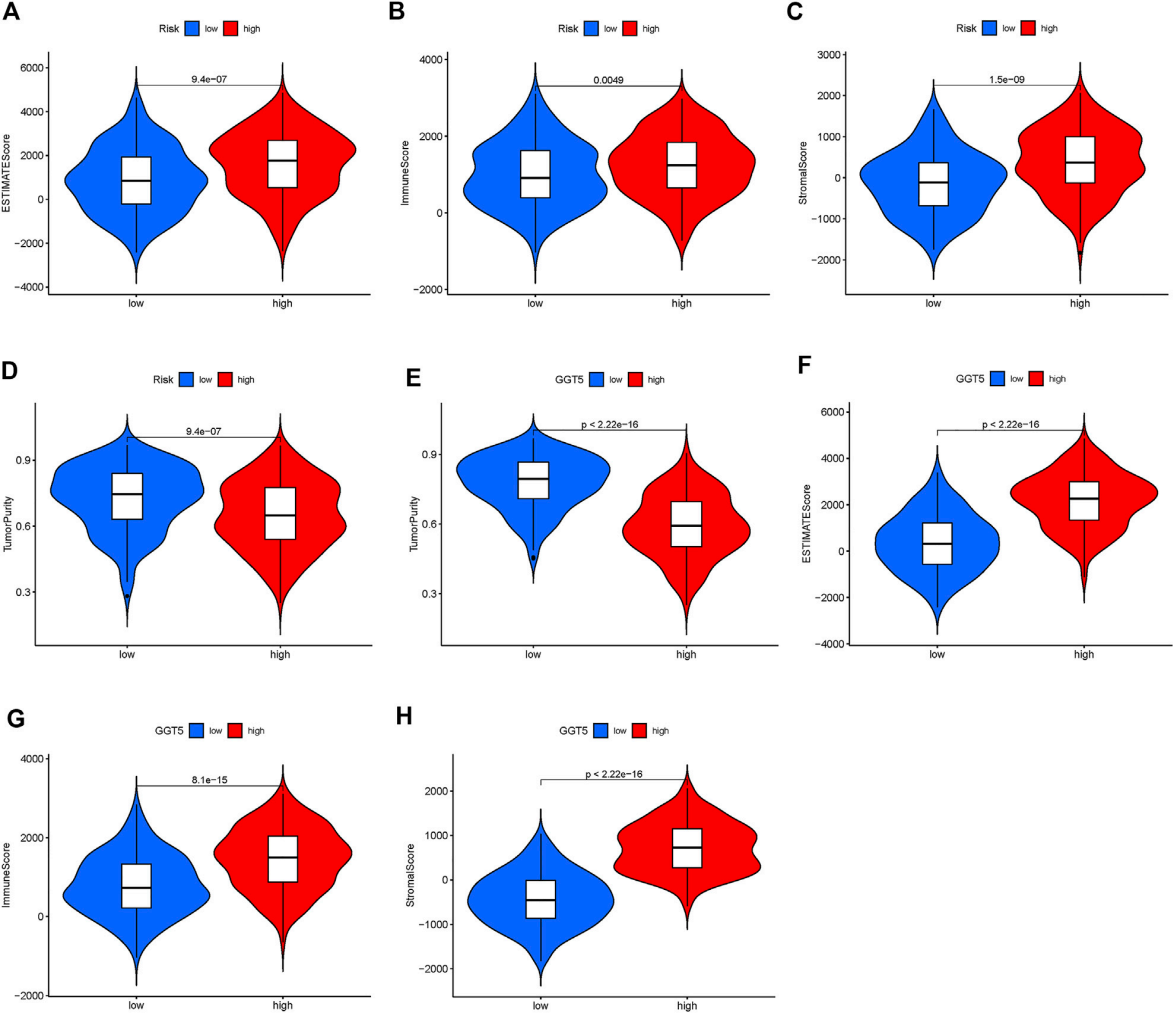
Difference analysis of tumor microenvironment in a different risk group and GGT5 expression group. **(A–D)**: difference analysis of tumor microenvironment in different risk groups; **(E–H)**: difference analysis of tumor microenvironment in different GGT5 expression groups. All of the scores are higher in the high-risk group and high GGT5 expression group, which indicates lower purity of tumors. (*p* < 0.05).

TIME is a major determinant of tumorigenesis and cancer progression, and its heterogeneity can affect cancer prognosis and treatment (Li et al., 2021). To this end, we analyzed the expression levels of the immune checkpoint PD-L1 in the high- and low-risk STAD patients and found that PD-L1 was up-regulated in the high-risk group (*p* = 0.049, Figure 8K). Furthermore, the TIMER database (http://timer.cistrome.org/) showed that PD-L1 expression was positively correlated to *GGT5* expression and negatively correlated to tumor purity (Figure 8B). By analyzing the correlation between immune cells and *GGT5* expression level, the relationship between *GGT5* expression level and immune infiltration was obtained. As shown in Figures 8E–J, the infiltration ratios of M2 macrophages and resting dendritic cells were positively correlated to the expression level of *GGT5* and the risk score (R > 0 and *p* < 0.05), while the infiltration ratio of follicular T helper cells was negatively correlated with both parameters (R < 0 and *p* < 0.05). Thus, a higher expression of *GGT5* was associated with a higher degree of immune cell infiltration in STAD, and *GGT5* ^high^ patients might be more sensitive to immune checkpoint therapy. Consistent with this hypothesis, most immune checkpoints were significantly up-regulated in the *GGT5* ^high^ group (Figure 8L, *p* < 0.05), with the exception of the human endogenous retrovirus-H long terminal repeat-associating protein 2 (HHLA2). Furthermore, the stromal score, immune score and ESTIMATE score were higher, and the tumor purity was lower in the *GGT5* ^high^ group compared to the *GGT5* ^low^ group (Figures 9E–H). Analysis of the TME in the different risk groups revealed that the stromal score, immune score and estimate score were also higher, while tumor purity was lower, in the high-risk group compared to the low-risk group. Taken together, the results suggest that the *GGT5* ^high^ STAD patients might benefit more from immunotherapy.

### Gene ontology and Kyoto Encyclopedia of Genes and Genomes pathway analyses

**FIGURE 10:**
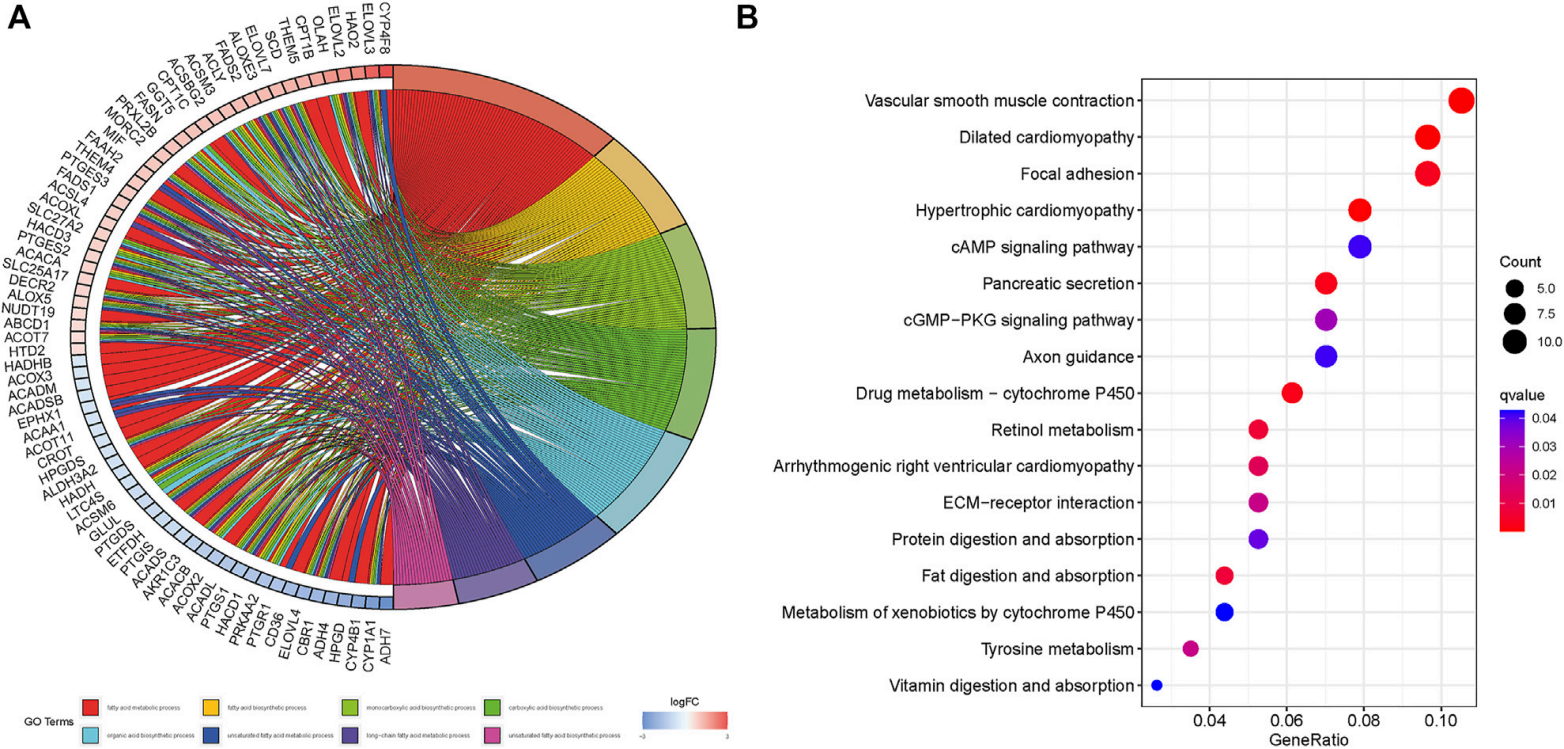
GO and KEGG enrichment of differential risk genes associated with fatty acid metabolism. **(A)**: GO analysis. **(B)**: KEGG analysis.

The genes that were differentially expressed between the high- and low-risk groups were functionally annotated by the GO and KEGG analyses using the DAVID (https://david.ncifcrf.gov/) online tool. According to GO analysis, the significantly enriched pathways relating to molecular functions (MF) were fatty acid metabolism, carboxylic acid biosynthesis, and organic acid biosynthesis pathways; the significantly enriched pathways relating to cellular component (CC) were peroxisome, microbody and mitochondrial matrix pathways; the significantly enriched pathways relating to biological processes (BP) were Oxidoreductase activity of acting on CH-OH group, the group of donors of NAD or NADP as acceptor and acyltransferase activity pathways. (Figure 10A). KEGG analysis showed that the vascular smooth muscle contraction, dilated cardiomyopathy, focal adhesion (FA), hypertrophic cardiomyopathy and cAMP signaling pathways were significantly enriched (Figure 10B).

## Discussion

STAD is one of the most frequently diagnosed malignancies and is associated with high mortality worldwide (Torre et al., 2015). Given the complex pathogenesis of STAD, surgery is considered to be the only radical cure; however, it can easily lead to serious complications such as anastomotic leakage, intestinal obstruction, and early recurrence, which can worsen the prognosis and reduce the OS of the patients (Van Cutsem et al., 2016; Zong et al., 2016; Li et al., 2017). In recent years, several immunotherapy or targeted therapy have been developed for STAD (Tarnawski et al., 2005; Wang et al., 2020b; Wang et al., 2021); however, patients could rarely benefit from these therapies, primarily due to the development of drug resistance. Multiple mechanisms drive therapeutic resistance in tumor patients, such as epigenetic and genetic dysregulation, altered signaling pathway and metabolic reprogramming (Cheetham et al., 2013). Of these mechanisms, metabolic reprogramming, in particular, is a hallmark of cancer; it can promote cancer cell proliferation and metastasis, thus contributing to malignant progression (Kroemer and Pouyssegur, 2008; Jiang et al., 2019). In addition, metabolic reprogramming in the TME can affect immune cell infiltration and function, eventually compromising immunotherapeutic efficacy (Pavlova and Thompson, 2016). Studies show that solid tumors can secrete large amounts of fatty acids, resulting in a fatty acid-rich TME. Furthermore, genes involved in lipogenesis are frequently up-regulated in prostate, colonic, ovarian, liver, lung, and other cancers (Swinnen et al., 2006; Wei et al., 2020). Therefore, drugs targeting fatty acid and other metabolic pathways might be effective in the treatment of some cancers (Piccinin et al., 2019).

In this study, we identified the FAM-related genes closely related to the prognosis of STAD, of which 4 were differentially expressed between the STAD tumor and adjacent normal tissues and may function as oncogenes or tumor suppressors. The genes up-regulated in the tumor tissues included ATP citrate lyase (ACLY) and monoamine oxidase A (MAOA). While ACLY expression is associated with advanced stage and prognosis in gastric adenocarcinoma (Qian et al., 2015), MAOA expression can promote the proliferation and metastasis of human gastric tumor cells by inducing mitochondrial dysfunction and aerobic glycolysis (Chen et al., 2020). Furthermore, reprogramming of FAM pathways plays an important role in the TME and influences cancer progression and therapeutic efficacy (Siddiqui and Glauben, 2022).

We constructed a prognostic model based on the FAM-related genes, and the patients were stratified into the low- and high-risk groups based on the risk score of these genes. The low-risk group had higher survival rates compared to the high-risk group in both the test and training datasets. A nomogram was then developed using the FAM-related prognostic signature and age to provide a quantitative means for prognosis prediction of STAD. The nomogram predicted the OS of patients with reasonable accuracy. The nomogram was independent of other clinical factors and could be applied to different clinical groups.

To assess the impact of the FAM-related genes on immune infiltration, we analyzed the immune cell scores in the two groups which were classified based on hierarchical clustering. The naïve B cells and resting memory CD4^+^ T cells were predominant in Group 2, and the CD8^+^ T cells were predominant in Group 1. Given that Group 2 corresponded to a high-risk score and poor prognosis, this finding indicates that the infiltration of naïve B cells and resting CD4^+^ memory T cells in the TME of STAD may portend a worse prognosis. This is consistent with the study by Zhao et al., who showed that naïve B cell infiltration in STAD tumors correlates with tumor metastases and fully functional regulatory activity against human stomach adenocarcinoma immunity (Zhao et al., 2021). Wu et al. suggested that resting CD4^+^ memory T cells cannot mount a sufficient immune response against STAD, and increased infiltration of these immune cells is detrimental to patient prognosis (Wu et al., 2021a). We also found that the immune scores were higher in the high-risk group, indicating lower tumor cell purity and more immune cell infiltration. Studies have shown that the TIME of metastatic tumors is less immunocompetent compared to that of primary STAD, which may help establish reliable prognostic signatures on the basis of stromal and immune components (Wang et al., 2019). The results of this study supported our hypothesis that immune cell infiltration in the TME affected the prognosis of patients with STAD.

Through GEPIA database analysis, we found that *GGT5* was an independent prognostic factor for STAD. *GGT5* was highly expressed in the STAD tumor tissues compared to adjacent normal tissues, and correlated with worse DFS and OS, in line with previous studies (Ye et al., 2021; Huang et al., 2022). Furthermore, *GGT5* overexpression in the tumor tissues was positively correlated with PD-L1 expression and CD8^+^ T cell infiltration. PD-L1, encoded by the CD274 gene, is a major co-inhibitory checkpoint that represses T cells (Ai et al., 2020). Multiple tumors overexpress PD-L1 and use the PD-L1/PD-1 signaling to evade T cell-mediated immune killing. Immunotherapies that target the PD-1/PD-L1 axis are effective against various cancers and have shown encouraging results in patients with advanced cancers. It is recognized as the gold standard for developing new immune checkpoint blockade (ICB) and combination therapies (Cha et al., 2019). In this study, we found that most immune checkpoints were up-regulated in the STAD tissues with high *GGT5* expression. One study showed that *GGT5* is highly expressed in cancer-associated fibroblasts (CAFs) derived from lung adenocarcinoma tissues, and contributes to cancer cell survival and drug resistance (Wei et al., 2020). According to the American Joint Committee on Cancer (AJCC) staging manual for STAD, advanced tumor stage is associated with a worse prognosis (In et al., 2017). *GGT5* was highly expressed in stages 3, T2, T3 and T4, as well as in stages II, III and IV tumors. These findings suggest that *GGT5* plays an active role in STAD progression, although its function and mechanism are not completely clear.

To further explore the potential FAM-related mechanisms in the occurrence and development of STAD, we functionally annotated the FAM-related prognostic genes by GO and KEGG analyses. GO analysis revealed that the fatty acid metabolism, carboxylic acid biosynthesis, organic acid biosynthesis, peroxisome, microbody and mitochondrial matrix, oxidoreductase activity and acyltransferase activity pathways were enriched, all of which have been correlated with cancer development (Dahabieh et al., 2018; Islinger et al., 2018; Bian et al., 2019). KEGG analysis further showed that the vascular smooth muscle contraction, dilated cardiomyopathy, focal adhesion (FA), hypertrophic cardiomyopathy and cAMP signaling pathways were enriched. FA is a membrane-related macromolecule assembly that links actin cytoskeleton to the extracellular matrix through integrin. It plays an important role in maintaining cellular tension and signal transduction for cell survival. Recent studies have shown that FA-related structural molecules also regulate the epithelial-mesenchymal transition (EMT) of tumor cells, and promote tumor invasion and metastasis (Wu et al., 2021b; Lin et al., 2022). One study showed that the activation of FA by cAMP-FAK signaling can promote prostate cancer invasion (Cheng et al., 2018). Furthermore, there is considerable evidence linking the cAMP signaling pathway and cancer progression (Ma et al., 2019; Schernthaner-Reiter et al., 2020). GSEA further identified the hypertrophic cardiomyopathy pathway, which was also revealed by the GO and KEGG analyses. Given the paucity of research on the signaling pathways involved in STAD progression, our findings provided valuable insights for exploring new directions in developing novel diagnostic and therapeutic methods.

## Conclusion

We established a prognostic FAM-related gene signature model for STAD, along with a predictive nomogram based on the 7-gene risk score and age of the patient. The overexpression of *GGT5,* in particular, was associated with worse prognosis, higher PD-L1 levels, and increased immune cell infiltration. Thus, STAD patients with high levels of *GGT5* in the tumors might be more responsive to immune checkpoint blockade and other immunotherapies.

## Data Availability

The datasets presented in this study can be found in online repositories. The names of the repository/repositories and accession number(s) can be found in the article/Supplementary Material.

## Abbreviations

STAD, stomach adenocarcinoma; TIME, tumor immune microenvironment; TME, tumor microenvironment; DFS, disease-free survival; TCGA, The Cancer Genome Atlas; KEGG, Kyoto Encyclopedia of Genes and Genomes; FAM, fatty acid metabolism; GSEA, Gene Sets Enrichment Analysis; GO, Gene Oncology; OS, overall survival; GGT5, gamma-glutamyltransferase 5; ICB, immune checkpoint blockade; MAOA, monoamine oxidase A; ACLY, ATP citrate lyase; OLAH, oleoyl-ACP hydrolase; PON1, paraoxonase 1; THEM5, thioesterase superfamily member 5; ABCD1, ATP binding cassette subfamily D member 1.
