# Supplementary material for: Classification of stomach adenocarcinoma based on fatty acid metabolism-related genes frofiling
Source: Front Mol Biosci. 2022 Aug 26;9:962435. doi: 10.3389/fmolb.2022.962435 (PMC9461144; doi:10.3389/fmolb.2022.962435)
Supplement: Supplementary file 1 [file Table1.DOCX]

**Table S1. Basic clinical information of 187 STAD patients in training set.**

| **Variables** | **Training set**  **(n=187)** |
| --- | --- |
| Age | 65.39±10.98 |
| Gender  Female  Male | 72(38.5%)  115(61.5%) |
| Tumor Grade  G1&G2  G3&G4  Unknow | 77(41.18%)  106(56.68%)  4(2.14%) |
| Pathologic Stage  I&II  III&IV  Unknow | 87(46.52%)  89(47.59%)  11(5.89%) |
| AJCC-T  T1  T2  T3  T4  Unknow | 11(5.88%)  45(24.06%)  79(42.25%)  49(26.20%)  3(1.61%) |
| AJCC-N  N0  N1-N3  Unknow | 58(31.02%)  122(65.24%)  7(3.74%) |
| AJCC-M  M0  M1  Unknow | 165(88.24%)  13(6.95%)  9(4.81%) |

Values are mean ± standard deviation or n (%).
