# Supplementary figures and images for: Classification of stomach adenocarcinoma based on fatty acid metabolism-related genes frofiling
Source: Front Mol Biosci. 2022 Aug 26;9:962435. doi: 10.3389/fmolb.2022.962435 (PMC9461144; doi:10.3389/fmolb.2022.962435)

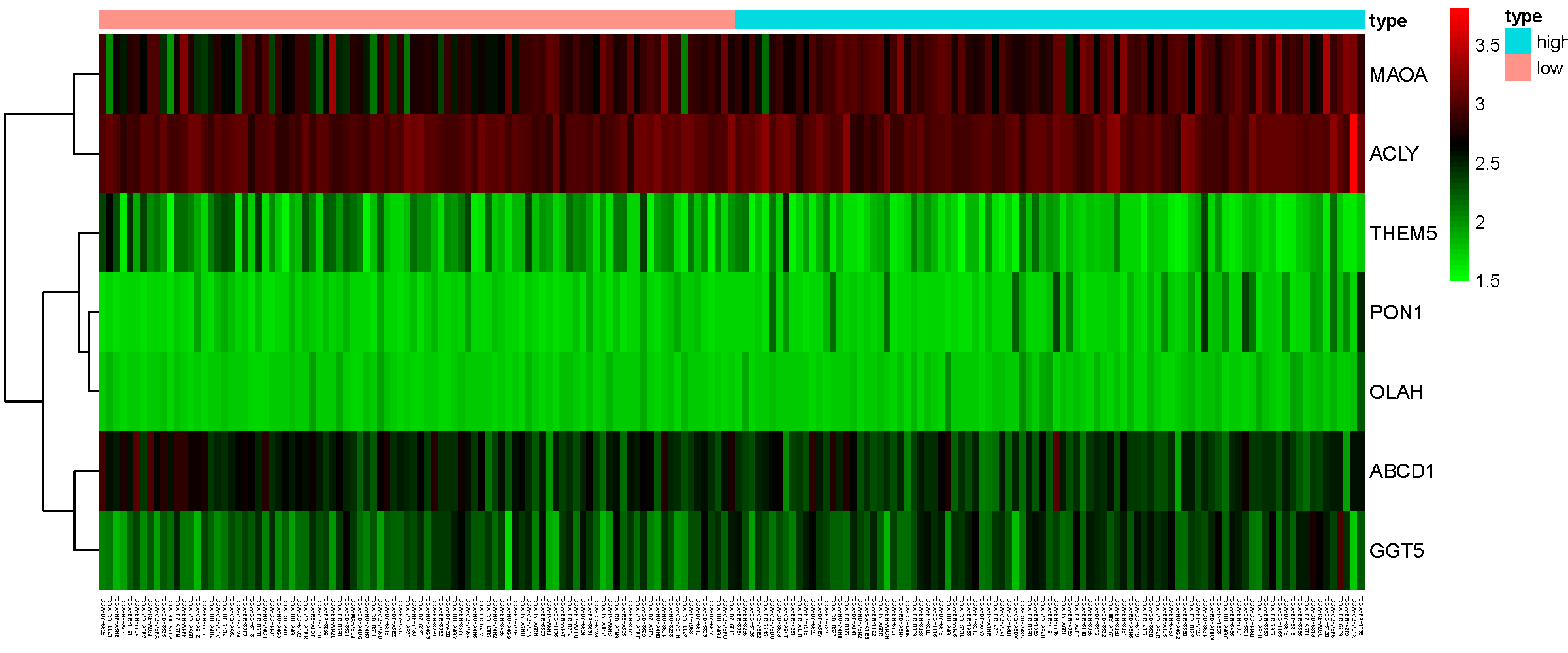

Supplement: Supplementary file 2 [file Image1.TIF]

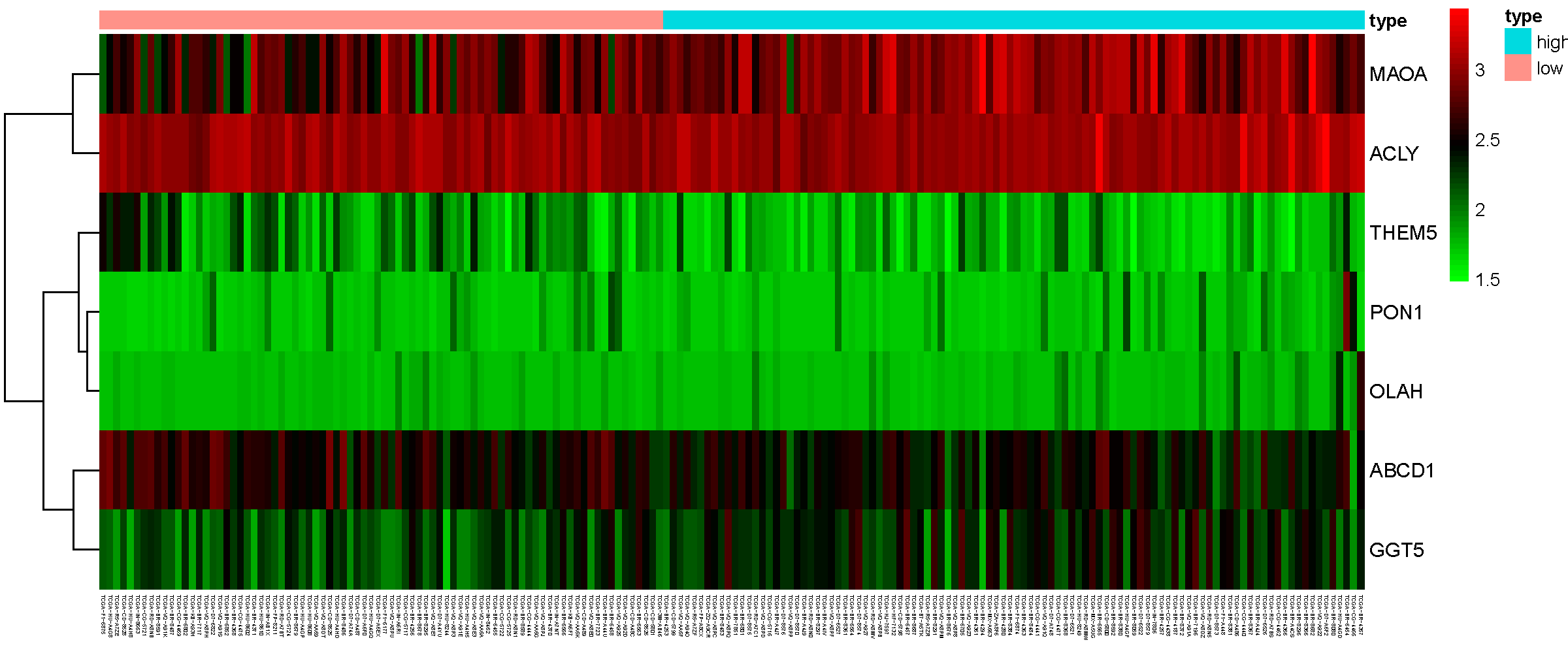

Supplement: Supplementary file 4 [file Image2.TIFF]
