# Supplementary material for: Classification of stomach adenocarcinoma based on fatty acid metabolism-related genes frofiling
Source: Front Mol Biosci. 2022 Aug 26;9:962435. doi: 10.3389/fmolb.2022.962435 (PMC9461144; doi:10.3389/fmolb.2022.962435)
Supplement: Supplementary file 3 [file Table2.DOCX]

**Table S2. Basic clinical information of 184 STAD patients in testing set.**

| **Variables** | **Testing set**  **(n=184)** |
| --- | --- |
| Age | 66.23±11.61 |
| Gender  Female  Male | 61(33.15%)  123(66.85%) |
| Tumor Grade  G1&G2  G3&G4  Unknow | 67(36.41%)  112(60.87%)  5(2.72%) |
| Pathologic Stage  I&II  III&IV  Unknow | 74(72.6%)  100(21.6%)  10(5.8%) |
| AJCC-T  T1  T2  T3  T4  Unknow | 7(3.80%)  33(17.93%)  88(47.83%)  51(27.72%)  5(2.72%) |
| AJCC-N  N0  N1-N3  Unknow | 50(27.17%)  123(66.85%)  11(5.98%) |
| AJCC-M  M0  M1  Unknow | 163(88.59%)  12(6.52%)  9(4.89%) |

Values are mean ± standard deviation or n (%).
